# Impact of surface treatments on the photocatalytic performance of anodic aluminum oxide templates

**DOI:** 10.1038/s41598-025-98635-3

**Published:** 2025-04-29

**Authors:** Carina Hedrich, Robin R. Petit, Matthias M. Minjauw, Anna R. Burson, Stefanie Haugg, Kaline P. Furlan, Christophe Detavernier, Jolien Dendooven, Robert H. Blick, Robert Zierold

**Affiliations:** 1 Center for Hybrid Nanostructures, Universität Hamburg, Luruper Chaussee 149, 22761 Hamburg, Germany; 2 Integrated Ceramic-based Materials Systems Group, Hamburg University of Technology (TUHH), Denickestraße 15, 21073 Hamburg, Germany; 3 CoCooN Group, Department of Solid State Sciences, Ghent University, Krijgslaan 281/S1, 9000 Ghent, Belgium; 4 Karlsruhe Institute of Technology (KIT), Institute for Applied Materials – Ceramic Materials and Technologies (IAM-KWT), Haid-und-Neu-Strasse 7, 76131 Karlsruhe, Germany

**Keywords:** Anodic aluminum oxide, Photocatalysis, Surface chemistry, XPS, Nanostructured templates, Materials for energy and catalysis, Nanoscale materials, Structural materials, Energy science and technology, Nanoscale materials

## Abstract

Nanostructured materials receive great interest nowadays due to their unique properties, increased surface areas, and superior performances with associated reduced material usage. However, directly nanostructuring functional materials themselves can be technologically challenging. Hence, nanostructured substrates such as anodic aluminum oxide (AAO) can serve as templates for depositing active materials. The templates’ chemical stability is crucial for accurately assessing functional materials. Since AAO structures incorporate electrolyte ions during anodization, their chemical stability is influenced by the subsequent processing after anodization. This work investigates the effect of various post-anodization modifications on the surface chemistry and photocatalytic performance of bare AAO structures. Treatments with H_2_O_2_ or H_3_PO_4_ can stabilize the photocatalytic performance of the AAO templates over consecutive measurements. XPS measurements indicate that such stabilization results from AAO surface chemistry alterations. Further, we explore the functionalization of these modified AAO templates with photocatalysts by atomic layer deposition. The photocatalytic performance of TiO_2_ as a chemically stable photocatalyst is not affected by the templates’ post-anodization treatment. In contrast, the performance of templates functionalized with Fe_2_O_3_ as an inherent chemically instable photocatalyst depends on the template stability. This work highlights the importance of chemically stable template materials for exploring the properties of new functional materials.

**Supplementary Information:**

The online version contains supplementary material available at 10.1038/s41598-025-98635-3.

## Introduction

Achieving high functionality while minimizing material usage is a pivotal aim in materials science^1,2^. Nanostructuring has emerged as a key strategy to meet this goal, enabling the tailoring of materials at the nanoscale to expand their functionalities^2,3^. Moreover, nanostructuring leads to significantly enlarged surface areas while the material usage is simultaneously reduced^2–5^. Accordingly, nanostructured materials are often utilized either directly or as templates for electronics, photonics, (photo-)catalysis, biomedicine, energy storage and conversion, environmental applications, and magnetic devices^2,3,6–8^. To identify the properties of functional materials deposited onto nanostructured templates, it is crucial to use a chemically stable structure as template^9,10^. Specifically, the template should not decompose or undergo any other chemical changes during the applications as it will be functionalized with an active material.

**Fig. 1:**
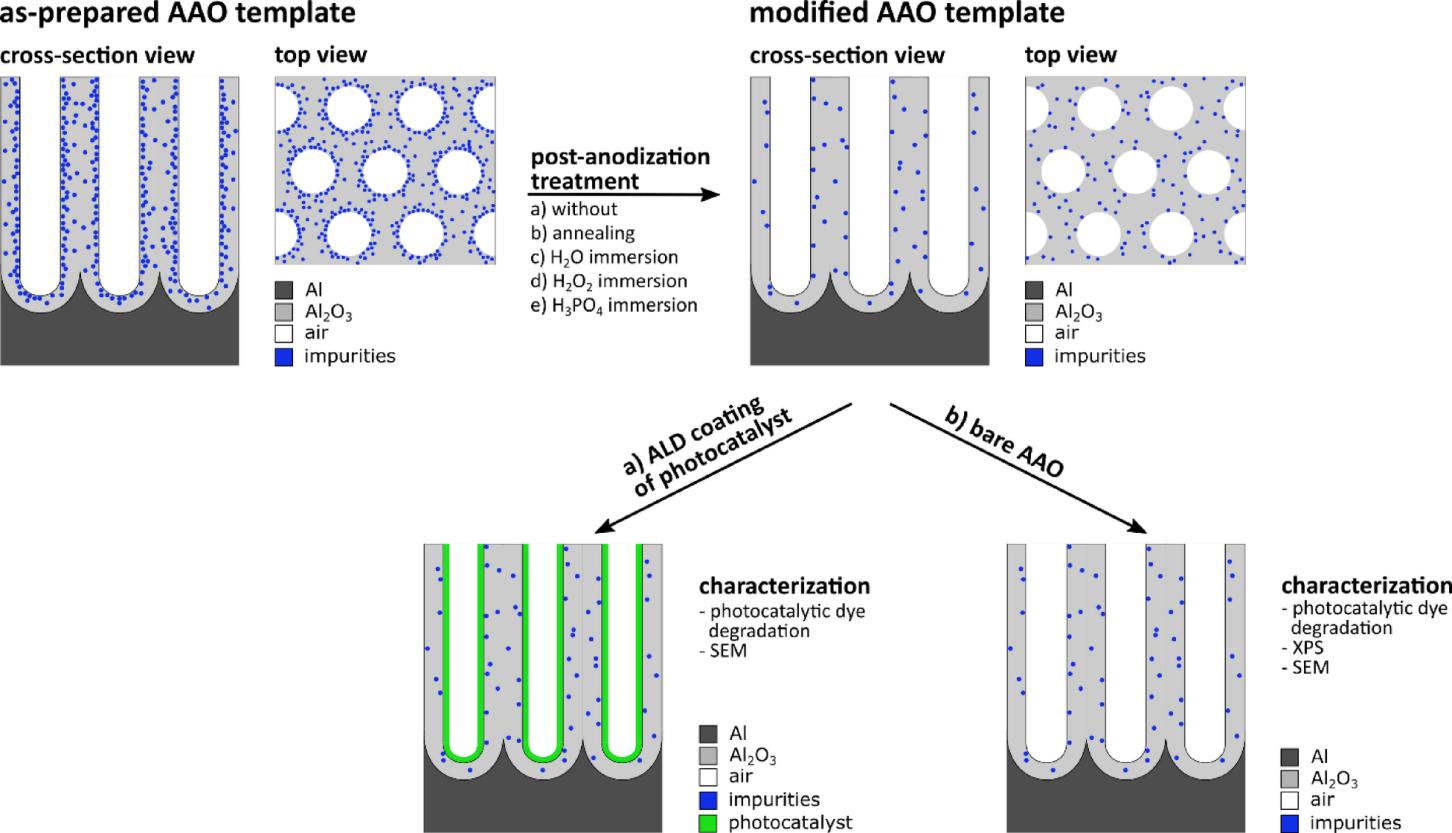
Schematic illustration of the AAO template structure, processing, and characterization. As-prepared AAO templates contain electrolyte impurities in their Al_2_O_3_ matrix. The impurities are incorporated during fabrication and their concentration is increasing from the Al_2_O_3_ matrix towards the Al_2_O_3_/air interfaces, i.e., the interfaces in contact with the electrolyte during anodization. Herein, post-anodization treatments are applied to modify the AAO templates’ surface chemistry by (partially) removing the impurities. The photocatalytic performance of (a) modified AAO templates with photocatalyst ALD coating and of (b) modified bare AAO templates is assessed by dye degradation. Moreover, the influence of the different post-anodization treatments on the surface chemistry of the AAO templates is characterized by X-ray photoelectron spectroscopy (XPS).

One prominent example of such nanostructured templates is anodic aluminum oxide (AAO) which has been used for many different applications such as optics, sensing, catalysis, energy storage, or electronics over the past years^6,11–14^. AAO consists of cylindrical pores inside an aluminum oxide (Al_2_O_3_) matrix produced by an electrochemical oxidation also known as anodization^13^. The pores are highly ordered in a hexagonal pattern due to a self-assembly process and are aligned perpendicular to the surface^8,15^. AAO provides a large flexibility as template structure, because the pore size, pore morphology, layer thickness, and surface functionalization can be widely tailored during or after the anodization^6,13,14^. Furthermore, due to the porous nature, AAO templates feature high surface areas which are beneficial for many applications, e.g., photo(electro)catalysis, solar cells, and batteries^6,7,14,16^. However, anodization is accompanied by contamination of the aluminum oxide matrix by the formation of oxygen vacancies in the aluminum oxide and through the incorporation of anions of the utilized acidic electrolyte^17–21^. The contamination percentage is increasing from the inner side of the pores towards the outer part, i.e., the one in contact with the electrolyte during anodization as schematically shown in Fig. 1. These contaminations are inevitable and they can cause undesired properties of the AAO template such as high photoluminescence activity^20–24.^ Moreover, these contaminations could be dissolved when exposed to or reacted with aqueous solutions which are applied, for example, to functionalize the AAO surface. Consequently, such dissolution processes may alter the intrinsic properties of AAO as a template material. Furthermore, besides chemical impurities, oxygen vacancies present in the AAO matrix are also known to cause photoluminescence of the structure^20–22,24,25^. Healing of these defects can be attempted by tailored annealing processes. Note, since AAO often serves as a template structure and thus is coated with functional materials, it is essential that the alumina surface is stable against the required treatments^8,26,27^. On the one hand, the surface chemistry has to be modified by depositing a material of choice onto the AAO template. On the other hand, the surface chemistry should not be altered in a way that affects the properties of the AAO template structure. Chemically stable templates non-interacting with the coating allow for investigating functional materials – even those which are prone to decompose during their application. The influence of AAO’s surface chemistry onto its applicability as template structure can for example be studied for photocatalytic applications. Photocatalysis is based on the induction of chemical reactions by irradiation with light and it offers a plethora of possible applications such as water or air purification, hydrogen production, self-cleaning surfaces, or energy conversion^4,28–32^. Since photocatalytic reactions occur at the surface of materials, an increment of the surface area corresponds to an increase of the reactions per time period potentially leading to a more efficient process^2–5^. Thus, AAO structures are a great choice of template structure for photocatalysts due to their high surface area, scalable and comparably inexpensive fabrication, and versatile functionalization possibilities.

This study explores the influence of different post-anodization treatments of AAO templates on their properties with the main goal of obtaining a stable and robust surface chemistry for the exemplary application of photocatalysis. Post-anodization treatments – namely immersion in hydrogen peroxide (H_2_O_2_) and immersion in phosphoric acid (H_3_PO_4_) – are applied prior to functionalization with titanium dioxide (TiO_2_) or iron (III) oxide (Fe_2_O_3_) as a photocatalyst by atomic layer deposition (ALD) and are compared to as-prepared AAO templates functionalized with the respective photocatalyst. Due to sequential, self-limiting reactions between precursors in the gas phase, and solid substrate surfaces, ALD allows for complete conformal coating of the substrate – here AAO – while simultaneously controlling the deposited film thickness in the sub-nanometer range^33–36^. TiO_2_-coated structures showed stable photocatalytic performances over three measurements for all investigated post-anodization modifications, suggesting little to no influence by the post-anodization treatments. In contrast, the photocatalytic activity of Fe_2_O_3_-functionalized samples is stable for AAO templates immersed in H_2_O_2_ or H_3_PO_4_ but decreases within the subsequent measurements for the as-prepared AAO template. This observed behavior is probably caused by modifications of the AAO surface chemistry depending on the respective post-anodization treatment. To reveal the influence of the surface treatments on the photocatalytic properties, the photocatalytic performances of bare post-anodization modified AAO templates without additional ALD coating (Fig. 1) were investigated. Furthermore, additional surface modifications were tested. In detail, immersion of AAO templates into water (H_2_O), H_2_O_2_, or H_3_PO_4_, and thermal annealing are applied as post-anodization treatments and compared to an as-prepared AAO template. The effect of the different modifications on the AAO surface chemistry is examined by X-ray photoelectron spectroscopy (XPS). The results obtained in this work shed light on modifying the surface properties of AAO templates driven by post-anodization modifications.

## Results and discussion

### Properties of ALD-functionalized AAO

Highly ordered AAO structures featuring a pore length of about 30 μm and a pore diameter of 25 nm were fabricated by anodization. Herein, the photocatalytic performance of post-anodization treated AAO templates is assessed by the degradation of MB as a model pollutant of water. ALD-coating of photocatalyst materials onto AAO templates exposed to hydrogen peroxide (H_2_O_2_) or phosphoric acid (H_3_PO_4_) after the anodization reveals the importance of the post-anodization treatment for the photocatalytic performance and stability of the photocatalyst. On the one hand, a chemically stable photocatalyst – here TiO_2_ – is not significantly affected by the post-anodization treatment. On the other hand, an inherent chemically instable photocatalyst – here Fe_2_O_3_ – is known to get degraded through redox reactions and photocorrosive processes and shows decreasing performance over subsequent measurements. Note, the as-deposited ALD-grown TiO_2_ and Fe_2_O_3_ films are amorphous and are used without further modification to prevent additional effects on the AAO template structure.

**Fig. 2:**
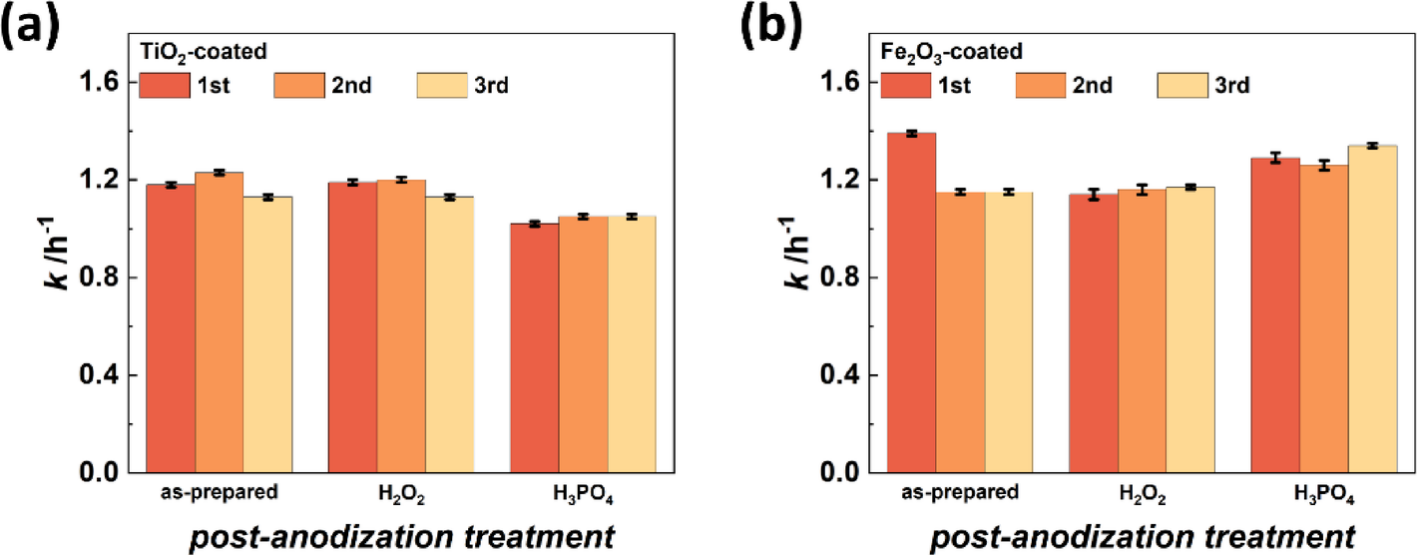
Photocatalytic performances of post-anodization modified AAO templates that were afterwards coated with a photocatalyst by ALD. (a) TiO_2_-functionalized samples show stable photocatalytic activities for all three post-anodization treatments over three consecutive measurements. The mean photocatalytic activities are 1.18 ± 0.05 h^-1^, 1.17 ± 0.04 h^-1^, and 1.04 ± 0.02 h^-1^ for as-prepared, H_2_O_2_-treated, and H_3_PO_4_-treated templates, respectively. (b) Coating Fe_2_O_3_ as a chemically less stable photocatalyst onto AAO templates demonstrates a dependence of the photocatalytic performance stability on the previously applied treatment. The Fe_2_O_3_-coated templates feature mean activities of 1.23 ± 0.14 h^-1^ (as-prepared), 1.16 ± 0.02 h^-1^ (H_2_O_2_-treated), and 1.30 ± 0.04 h^-1^ (H_3_PO_4_-treated).

TiO_2_-functionalized structures presented stable photocatalytic properties regardless the surface treatment as depicted in Fig. 2a. The 5 nm thick amorphous TiO_2_ coatings deposited onto the AAO templates are chemically very stable and thus, it is expected that they are not strongly influenced by the underlying template^37^. Fig. 2a shows stable photocatalytic activities of TiO_2_-functionalized AAO templates during three consecutive measurements for all three investigated templates, namely as-prepared AAO, H_2_O_2_-treated AAO, and H_3_PO_4_-treated AAO. The mean activities of as-prepared and H_2_O_2_-treated samples are very similar (1.18 ± 0.05 h^-1^ and 1.17 ± 0.04 h^-1^). However, the structure immersed in H_3_PO_4_ solution features a lower photocatalytic activity of 1.04 ± 0.02 h^-1^. The reasons for this behavior are discussed in details when presenting the XPS measurements.

Meanwhile, the photocatalytic performance of 5 nm amorphous Fe_2_O_3_-functionalized AAO shows a strong dependence on the post-anodization treatment as shown in Fig. 2b. We exclude photocorrosion of the Fe_2_O_3_ layer as a possible explanation since an ultra-thin Al_2_O_3_ coating of 2 ALD cycles was deposited onto the structures^38^. By analyzing and comparing the data from Fe_2_O_3_ to TiO_2_, three key findings have been revealed: First, the photocatalytic performance of Fe_2_O_3_-functionalized AAO is very stable over three measurements for structures treated with H_2_O_2_ or H_3_PO_4_ featuring mean activities of 1.16 ± 0.02 h^-1^ and 1.30 ± 0.04 h^-1^, respectively. In contrast, a strong decrease of the activity after the first measurement is observed for an as-prepared AAO template. This performance loss resembles in a high standard deviation (1.23 ± 0.14 h^-1^). Second, and at the same time, the mean activity for H_3_PO_4_-treated AAO functionalized with Fe_2_O_3_ is significantly higher than for a treatment with H_2_O_2_. Third, this observation is in contrast to the TiO_2_-coated AAOs, where the H_3_PO_4_-treatment reveals the lowest activity.

Electrolyte ions present in the Al_2_O_3_ matrix of the as-prepared AAO can potentially react with the surface when activated by charge carriers of the photocatalytic processes. In this way, the photocatalyst is chemically altered and the photocatalytic activity is influenced. EDX measurements of the TiO_2_-coated AAO templates demonstrated an almost constant Ti content independent of the surface treatment before ALD (Table S1) proving that the influence of the surface treatment onto the TiO_2_ ALD process is negligible. Due to charging effect, measurement of iron oxide was not straight forward, but we do not foresee any influence of the treatments onto the ALD growth. XPS measurements of the ALD-grown photocatalysts are shown in Figure S1. For the TiO_2_-functionalized AAO structure, the Ti_2p3/2_ peak and the Ti_2p1/2_ peak feature a spin-orbit splitting of 5.7 eV indicating the Ti(IV) oxidation state which is present in TiO_2_^39,40^. Moreover, based on the survey spectrum, the atomic percentages for Ti and O are present with the expected ratio for TiO_2_, when taking into account the O-bonds with C and Al. The Fe_2p3/2_ peak and the Fe_2p1/2_ peak of the Fe_2_O_3_-coated AAO structure are separated by 13.2 eV. In combination with the Fe_2p3/2_ peak position at a binding energy (B.E.) of 711 eV, low satellite peak intensities and the atomic percentages for Fe and O, such features were previously assigned to the Fe_2_O_3_ phase^41^.

The applied surface treatments and ALD coating did not influence the morphology of the straight pores of the AAO templates as depicted in the cross-section scanning electron microscope (SEM) images in Figure S2. Further, no morphology changes are observed when comparing the samples before and after the photocatalysis measurements. Top-view SEM images in Figure S3 confirm that the ALD-coated AAO templates do not present a visible change in the pore morphology or film structure before and after photocatalysis tests. Note that exploring new photocatalytic or other functional materials coated onto AAO templates necessitates a chemically stable and known template to accurately characterize the materials’ properties. Hence, we studied the influence of common post-anodization treatments on the photocatalytic performance of AAO templates to obtain a more detailed understanding of the surface chemistry changes which do not affect the pore morphology.

### Effect of post-anodization surface treatments on AAO templates

**Fig. 3:**
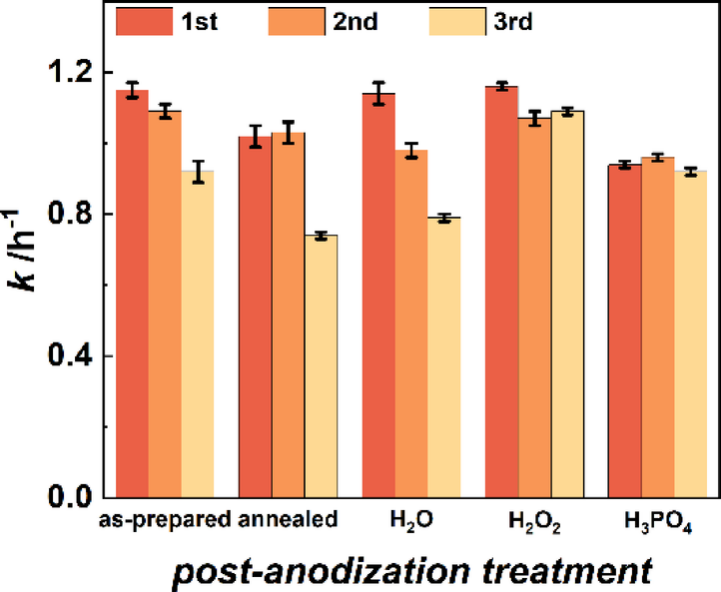
Photocatalytic performances of post-anodization modified AAO templates. The photocatalytic activities remain constant or decrease over three consecutive measurements depending on the applied treatment. Differences in the stability of the photocatalytic performances are also reflected in the mean value and standard deviation of the activity *k*. The modified templates feature the following mean activities: 1.05 ± 0.12 h^-1^ (as-prepared), 0.93 ± 0.16 h^-1^ (annealed), 0.97 ± 0.18 h^-1^ (H_2_O-treated), 1. 11 ± 0.05 h^-1^ (H_2_O_2_-treated), and 0.98 ± 0.02 h^-1^ (H_3_PO_4_-treated).

The influence of four different post-anodization treatments of AAO templates on their photocatalytic performance is investigated to identify treatments which stabilize the AAO properties. Taking frequently reported treatments of AAO templates such as pore diameter modifications into account, we assessed the effects of AAO thermal annealing, and immersion into H_2_O, H_2_O_2_, or H_3_PO_4_ compared to an as-prepared AAO template. As depicted in Fig. 1, these templates are referred to as bare AAO templates since they were exposed to post-anodization treatments but no further coating was applied. As reference, the obtained photocatalytic activity of a blank measurement (without sample) and a 20 nm Al_2_O_3_-coated planar Si wafer are shown in Figure S4. The presence of H_2_O_2_ in the analyte solution can also contribute to direct photolysis of MB. Hence, a low MB degradation rate of 0.52 ± 0.01 h^-1^ is observed even when no samples is tested. While the photocatalytic activity is decreasing for an as-prepared and annealed AAO template (Fig. 3) within three consecutive measurements (1.05 ± 0.12 h^-1^ and 0.93 ± 0.16 h^-1^, respectively), it can be stabilized by certain wet-chemical post-anodization treatments. Decreasing photocatalytic activities in consecutive measurements are typically caused by the change of the chemical state of the structures’ surface or by structural decomposition^42–44^. Since Al_2_O_3_ is a chemically and temperature stable material and no additional photocatalyst material is applied here, saturation or removal of contamination electrolyte ions are possible reasons for the decreasing activity. These species provide unsaturated sites within the alumina matrix and can therefore lead to undesired side-reactions where they react with other compounds or they can potentially be removed from the structure by dissolution in aqueous media^45^. The side-reactions occur until all accessible contaminations have either reacted to stable species or are removed from the Al_2_O_3_ matrix, for example by dissolving them in a pore filling medium. The H_2_O-treated AAO template exhibits a clearly decreasing photocatalytic activity within consecutive measurements as also indicated by an increased standard deviation (0.97 ± 0.18 h^-1^). In contrast, AAO templates immersed in H_2_O_2_ for 24 h or in H_3_PO_4_ for 1 min show the most stable photocatalytic performance over three measurements. Although the activity variation is slightly larger for the H_2_O_2_-treated samples (1. 11 ± 0.05 h^-1^) than for the H_3_PO_4_-treated sample (0.98 ± 0.02 h^-1^), its mean activity is higher.

**Fig. 4:**
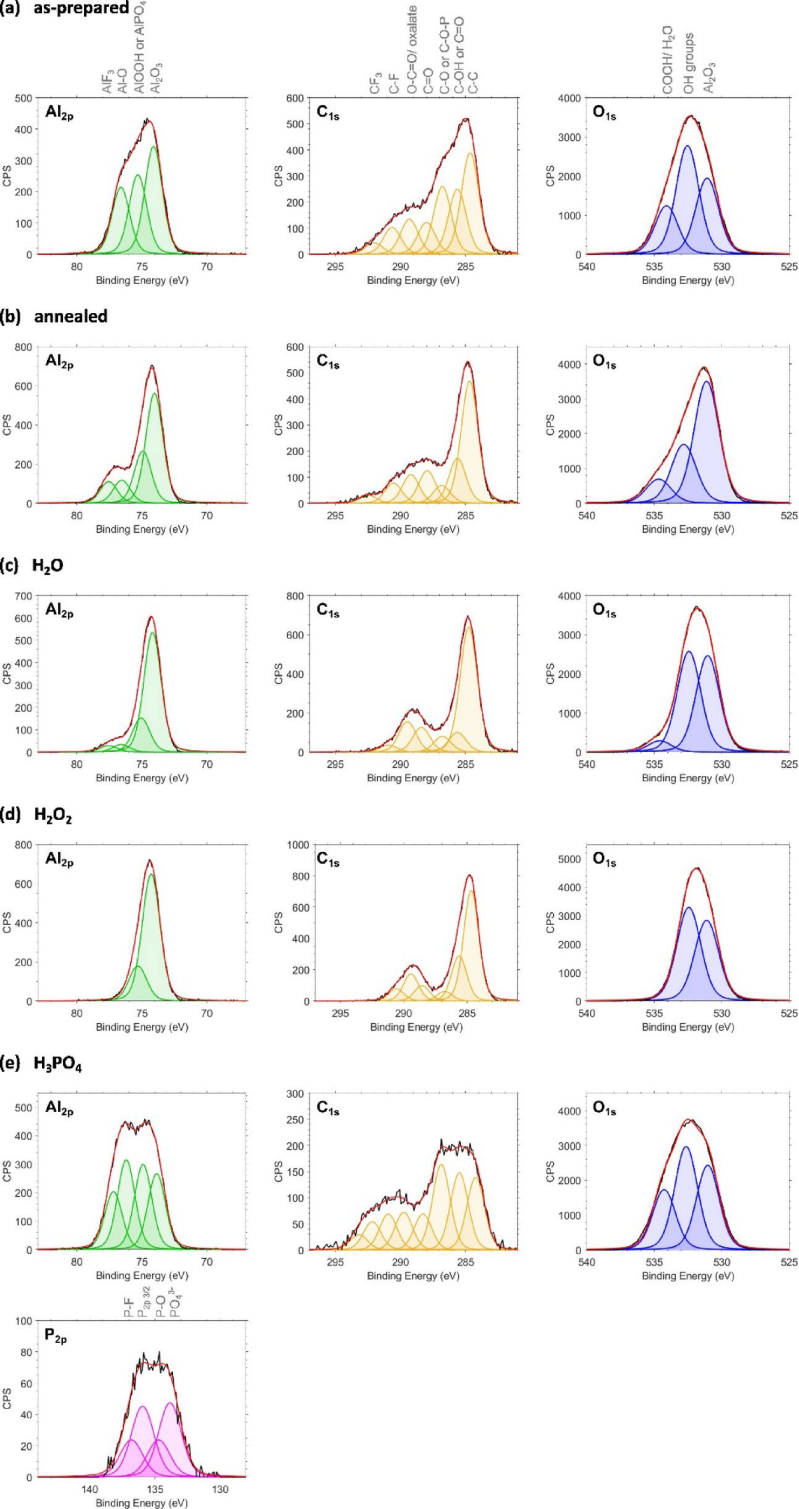
XPS measurements reveal the influence of different post-anodization treatments on the surface chemistry of AAO templates. The AAO templates are (a) as-prepared, (b) annealed at 450 °C for 1 h, (c) exposed to H_2_O for 24 h, (d) immersed into 30 wt% H_2_O_2_ for 24 h followed by H_2_O for 24 h, and (e) soaked into 5 wt% H_3_PO_4_ for 1 min.

The different absolute photocatalytic activities are hypothesized to originate from the different surface chemistry after post-anodization treatments. Our observation is in agreement with similar results which have previously been reported for TiO_2_ nanoparticles and for anodized TiO_2_^45,46^. Hence, we investigated the surface chemistry variations in detail with XPS measurements of the AAO structures after post-anodization treatments. The identified peak positions for all samples are summarized in Table S2 in the Supporting Information and the data analysis parameters are listed in Table S3. These XPS measurements demonstrate changes of the present surface groups and their ratios depending on the respective post-anodization treatment (Fig. 4). The as-prepared AAO template (Fig. 4a) features Al_2_O_3_, Al-OH groups, and O-Al-O bonds at the Al_2p_ peak corresponding to the Al_2_O_3_ matrix and expected hydroxy groups at the surface^6^. Different types of carbon-carbon and carbon-hydrogen bonds, carbon-oxygen bonds, and carbon-fluor bonds can be identified at the C_1s_ peak. The carbon-carbon and carbon-oxygen bonds are probably caused by incorporated oxalate ions. These ion species originate from the inherent structure of the produced AAO, i.e., electrolyte ions built into the aluminum oxide during the anodization process^13,19^. Hence, O-C = O, C-C, and C-H bonds at the AAO surface identified by the XPS measurements can be attributed to residues of oxalic acid (H_2_C_2_O_4_) which was used as electrolyte during the anodization process. For the O_1s_ peak of the as-prepared structure, Al_2_O_3_ bonds, OH groups, and COOH groups and adsorbed water molecules are observed. Note that for all discussed peaks, some bonds cannot be distinguished as their B.E.s are too close to each other. XPS survey spectra of the AAO templates modified with different surface treatments are depicted in Figure S5.

Post-anodization treatments can reduce the amount of contaminations as revealed by the XPS spectra. Thermal annealing predominantly removes -OH groups, -O bonds, and adsorbed H_2_O molecules from the surface as shown in Fig. 4b. Such behavior is in agreement with existing literature about the influence of annealing on the surface of Al_2_O_3_^19,22,24^. Thermal annealing was previously reported of being capable to reduce photoluminescence intensities of AAO templates due to the thermal decomposition of the incorporated electrolyte ions and saturation of oxygen vacancies during the annealing^22,24^. Such treatment affects the complete alumina matrix and thus, results in a large amount of electrolyte decomposition and vacancy saturation which can no longer contribute to photoluminescence-induced degradation of the dye. The -OH groups are not completely removed which might be caused by the comparable low annealing temperature of 450 °C, since Han et al. reported that annealing temperatures of at least 500 °C are necessary to remove -OH groups^24^. Although the subpeaks of the C_1s_ peak and the Al-OH contents are reduced for the sample annealed at 450 °C for 1 h, the photocatalytic activity could not be stabilized but instead shows a lower mean value. To prove further removal of -OH groups, oxalate incorporations, and carbon content, another AAO structure was annealed at 600 °C for 1 h and characterized by XPS. The spectra shown in Figure S6 and summarized in Table S4 demonstrate a reduction of -OH groups, Al-O bonds, and C content compared to the AAO structure annealed at 450 °C which is in good agreement to previous reports^19,22,24^. Immersing AAO templates in aqueous media could potentially dissolve oxalate ions incorporated into the AAO structure during anodization. XPS spectra of the AAO template soaked in H_2_O demonstrate a decreased Al-OH signal compared to the as-prepared sample (Fig. 4c). Moreover, the C-OH content and C-O bond signal are decreasing. Based on the XPS results, we assume that the H_2_O post-anodization treatment dissolves oxalic acid molecules which might then partially react with the AAO surface to form Al-oxalate. It was previously reported that treatments in aqueous solutions can lead to the dissociation of incorporated electrolyte ions in anodized metal oxides^45^. In this way, the amount of ions inside the AAO matrix in close vicinity to the surface gets reduced. A H_2_O treatment might be capable of removing electrolyte ions from the alumina matrix by dissolution, but the ions then present in solution cannot be further decomposed or chemically bound. A significant decrease in the photocatalytic performance observed for the H_2_O-immersed AAO templates over consecutive measurements could be caused by the partial dissolution of oxalate species from the matrix during the post-anodization treatment. Since these species could not be decomposed simply by the H_2_O treatment, they might physisorb at the surface and could potentially contribute to the photocatalytic activity. However, they are degrading during the photocatalytic reaction resulting in decreasing activities with increasing measurement numbers.

If the aqueous solution contains oxidizing species such as hydrogen peroxide (H_2_O_2_), the removed electrolyte ions can immediately be further decomposed. The destruction of oxalate species by H_2_O_2_ results in the complete oxidation of the ions to carbon dioxide (CO_2_)^47^. As depicted in Fig. 4d, immersion of AAO into H_2_O_2_ removes Al-OH groups and especially Al-O bonds from the surface as indicated by the Al_2p_ peak profile. While the content of O-C = O groups slightly increases in the C_1s_ peak, the Al_2_O_3_ and OH group signals in the O_1s_ peak also increase. Here, the sub-peak of COOH groups/ adsorbed water molecules is no longer present. This indicates that incorporated oxalic acid species are likely dissolved by this treatment and could potentially react with Al-OH and Al-O groups at the surface to form stable Al-oxalate complexes explaining the increasing Al_2_O_3_ content. In addition, H_2_O_2_ could act as an electron acceptor and thus accelerate the formation of Al-oxalate. Further, the present OH-groups are mainly bound to oxalate species. The photocatalytic activity stabilization for the H_2_O_2_ treatment is probably caused by the saturation of surface groups and removal of electrolyte ions from the surface vicinity as observed in the XPS measurements. The proposed formation of Al-oxalate results in a stable surface chemistry which is not affected by the solution utilized in the photocatalysis measurements or the photocatalytic reactions themselves, also displayed in Fig. 3 by a high mean photocatalytic activity with a low standard deviation. Moreover, no additional washing out of electrolyte ions can occur because the ions are already removed, decomposed, or saturated after the post-anodization treatment with an H_2_O_2_-containing aqueous solution.

H_3_PO_4_ is often applied for pore-widening of AAO templates by etching alumina^18,19^. During the alumina dissolution, this etching process simultaneously releases the incorporated electrolyte species from the structure^18^. Since aluminum ions are released into the solution by the etching, it is possible that they react with the dissolved electrolyte ions to form side-products such as aluminum oxalate. Figure 4e demonstrates decreasing peaks of O-C = O, C = O, and C-C/C-H bonds and a slight increase of the OH groups in the O_1s_ peak compared to the as-prepared sample. However, besides the hypothesized formation of Al-oxalate complexes, reactions of the phosphoric acid with Al ions are also possible. We assume that they result in the formation of Al-phosphate. Since the B.E.s for O = P, O-P, and Al-O-P groups (additional panel in Fig. 4e)) are similar to the aforementioned ones, it cannot be unambiguously distinguished. The Al_2p_ peak shows a similar ratio of both sub-peaks which was not observed for any of the other treatments. This might also be caused by the additional groups featuring similar binding energies, namely AlPO_4_ similar to Al(OOH). The additional P_2p_ peak also reveals the presence of phosphor containing groups. Specifically, PO_4_^3-^ and P-O-Al groups are detected. For samples immersed in H_3_PO_4_, the proposed formation of phosphor containing aluminum groups might reduce the overall photocatalytic activity due to changes in the dye adsorption at the AAO surface. Hence, these structures feature a lower mean activity after three photocatalysis measurements. However, the activity variation is even lower than for the H_2_O_2_-treated sample which probably originates from stabilization of the surface chemistry by the formation of Al-phosphate groups. The surface chemistry alterations by the different treatments do not affect the morphology of the AAO templates as demonstrated by cross-section SEM images (Figure S7). Moreover, the pore morphology is not altered during the photocatalysis measurement. This is also exemplarily shown by top-view SEM images of the H_2_O_2_-treated AAO template (Figure S8).

The photocatalysis measurements of the ALD-functionalized AAO templates presented in Fig. 2 can also be well explained by the results from the XPS study. TiO_2_-coating of as-prepared and post-anodization surface-modified AAO samples show stable photocatalytic activities over three measurements. The variations between the first and third measurements are very small and all tested templates benefit from the TiO_2_ functionalization by exhibiting increased photocatalytic activities compared to the uncoated counterparts depicted in Fig. 3. In detail, the TiO_2_ coating improved the photocatalytic performances by up to 12%. Note, the small increase in photocatalytic activity after TiO_2_ coating is associated with the utilized light source which emits mostly visible radiation that is not suitable to excite TiO_2_. The ratio of the photocatalytic performances when comparing the individual treatments remains constant – as-prepared and H_2_O_2_-modified templates demonstrate similar photocatalytic activities, but the H_3_PO_4_-treated sample shows a lower activity. This is in good agreement with the high chemical stability of TiO_2_^37^. Similarly, the constant photocatalytic activities of H_2_O_2_- and H_3_PO_4_-treated AAO templates are maintained after Fe_2_O_3_ functionalization. In contrast, as-prepared templates coated with Fe_2_O_3_ show decreasing activities over consecutive measurements. The activity decrease might be caused by the reaction of electrolyte ions in the as-prepared AAO structure with Fe_2_O_3_ when activated by charge carriers during the photocatalytic processes. This could induce chemical modification of Fe_2_O_3_ at the interface to the AAO template which could result in an activity decline over multiple measurements. Consequently, improving the chemical stability of the templates’ surface is beneficial for studying chemically less stable photocatalysts such as Fe_2_O_3_. Note that depositing Fe_2_O_3_ onto AAO templates modified with H_3_PO_4_ significantly boosts the photocatalytic performance compared to functionalizing a H_3_PO_4_-treated AAO template with TiO_2_. We believe that coating Fe_2_O_3_ onto the treated AAO template might not only result in the growth of Fe_2_O_3_, but might also lead to the formation of iron phosphate or iron hydroxy phosphate compounds based on the presence of phosphate groups at the interface between the surface modified AAO template and the ALD prepared Fe_2_O_3_ layer^48–50^. These materials also exhibit good photocatalytic activities as identified by previous studies^48–53^. Local formation of iron phosphate or iron hydroxy phosphate at the AAO/Fe_2_O_3_ interface might explain the significant performance increase when H_3_PO_4_-treated AAO templates are functionalized with Fe_2_O_3_ instead of TiO_2_.

## Conclusion

To sum up, post-anodization treatments of AAO templates modify their surface chemistry as revealed by XPS measurements. We found that functional groups originating from electrolyte ions incorporated into the AAO matrix during anodization can be reduced by different treatments. Specifically, the Al-OOH and Al-O groups as well as C = O are decreased while the Al_2_O_3_ content increased. Modifications of the surface chemistry by the tested treatments determine the photocatalytic behavior of the respective AAO template structures. Especially the stability of the photocatalytic activity over consecutive measurements can be enhanced when aqueous solutions containing H_2_O_2_ or H_3_PO_4_ are utilized. Functionalizing post-anodization modified AAO templates with TiO_2_ results in stable photocatalytic performances over consecutive measurements, because TiO_2_ itself is a chemically very stable material. Thus, it is not influenced by the surface chemistry of the underlying AAO template. Contrary, the photocatalytic stability of AAO templates functionalized with Fe_2_O_3_ – as a chemically less stable photocatalyst – is significantly affected by the post-anodization treatment. These results could assist the characterization of new functional materials in the future by employing chemically stable nanostructured templates, such as post-anodization modified AAO.

## Methods

### Materials

Aluminum (Al) chips (99.9997%, 0.05 mm thickness, 2 cm diameter) were received from Goodfellow GmbH (Germany). Perchloric acid (HClO_4_, CAS 7601-90-3), ethanol (C_2_H_5_OH, EtOH, CAS 64-17-5), isopropyl alcohol (C_3_H_8_O, IPA, CAS 67-63-0), oxalic acid (H_2_C_2_O_4_, CAS 144-62-7), chromium (VI) oxide (CrO_3_, CAS 1333-82-0), phosphoric acid (H_3_PO_4_, CAS 7664-38-2), hydrochloric acid (HCl, CAS 7647-01-0), copper (II) chloride dihydrate (CuCl_2_ · 2 H_2_O, CAS 10125-13-0), methylene blue (C_16_H_18_ClN_3_S, MB, CAS 122965-43-9), hydrogen peroxide (H_2_O_2_, CAS 7722-84-1), and titanium tetraisopropoxide (TTIP, CAS 546-68-9) were purchased from Sigma Aldrich (Germany) and used as received. Milli-Q water (> 16 MΩ cm, H_2_O) was utilized as ALD precursor and to prepare aqueous solutions. Ferrocene (C_10_H_10_Fe, Cp_2_Fe, CAS 102-54-5) was supplied by Alfa Aesar (Germany) and trimethylaluminum (C_3_H_9_Al, TMA, CAS 75-24-1) was purchased from Strem Chemicals (France). Nitrogen (6.0) and oxygen (5.0) were supplied by SOL (Germany) and Westfalen (Germany), respectively.

### Fabrication of AAO

AAO templates were produced by two-step anodization of aluminum. Prior to anodization, the Al chips were cleaned by immersion in IPA and H_2_O for 30 min, respectively, and were dried under nitrogen stream. The cleaned chips were electropolished in a HClO_4_/EtOH solution (1:4, v: v) at 20 V and 5 °C for 3 min. The first anodization step was conducted in aqueous H_2_C_2_O_4_ (0.3 M) at 40 V and 6 °C for 20 h. Afterwards, the formed nanoporous Al_2_O_3_ film was removed by etching in aqueous H_2_CrO_4_/H_3_PO_4_ solution (1.8 wt%/ 6 wt%) at 45 °C for 24 h. The second anodization step was conducted under the same conditions as the first one except the duration which was set to 10 h.

### Surface modification of AAO

Subsequent to the anodization, AAO templates were treated by different surface modification approaches while one sample was kept as-prepared without further treatment as reference. The surface treatments were as follows: (i) heat: annealed at 450 °C on a hotplate for 1 h in ambient atmosphere; (ii) polar solvent: immersed into H_2_O for 24 h and afterwards dried with N_2_ stream; (iii) chemical type I: immersed into H_2_O_2_ (30 wt%) for 24 h and H_2_O for another 24 h before it was dried under N_2_ stream; (iv) chemical type II: immersed into H_3_PO_4_ (5 wt%) at 45 °C for 1 min, rinsed and dried with a N_2_ stream. Annealing at 600 °C for 1 h was conducted in ambient atmosphere in a rapid thermal annealing oven OTF-1200X-4-RTP (MTI Corporation, USA). The heating rate was 5 °C/s and the base pressure was set to 200 Torr.

For functionalizing AAO templates with a photocatalyst, one as-prepared sample, one sample immersed in H_2_O_2_ (30 wt%) for 24 h and H_2_O for 24 h, and one sample exposed to H_3_PO_4_ for 1 min were coated with TiO_2_ or Fe_2_O_3_ and an ultra-thin Al_2_O_3_ protection layer by ALD, respectively. TiO_2_ coating was conducted in a GEMStar XT^™^ (Arradiance, USA) ALD reactor under stop-flow conditions at 150 °C and a N_2_ carrier gas flow of 30 sccm. TTIP heated to 80 °C and H_2_O at room temperature were pulsed for 0.1 s and 0.05 s, respectively. Exposure and purge times of 60 s and 120 s were applied for both half reactions. 96 cycles were applied to obtain a TiO_2_ thickness of 5 nm. Cp_2_Fe at 100 °C and O_3_ at room temperature were used as precursors in the Fe_2_O_3_ process which was operated in stop-flow mode at 200 °C in a home-built ALD system. Each Cp_2_Fe half-cycle consisted of 2 s precursor pulse, 60 s exposure, and 90 s pumping of the system. The O_3_ half-cycle was repeated twice within one complete ALD cycle and 0.08 s precursor pulse, 30 s exposure, and 90 s pump time were applied. Each sample was exposed to 179 cycles for Fe_2_O_3_ deposition resulting in a coating thickness of 5 nm. Subsequently, the samples were coated with an ultra-thin film of Al_2_O_3_ as protection layer to prevent photocorrosion of the Fe_2_O_3_ film^38^ Al_2_O_3_ deposition was conducted in stop-flow mode at 150 °C using TMA and H_2_O (both at room temperature) as precursors. Both half-cycles consisted of 0.05 s precursor pulse, 60 s exposure, and 90 s pumping. The samples were coated by 2 cycles of Al_2_O_3_.

### Composition and structural analysis of modified AAO

XPS measurements were conducted in a Theta Probe XPS instrument (Thermo Fisher Scientific Inc., USA) and the XPS data analysis was performed using the CasaXPS software package. A pass energy of 50 eV and 200 eV and a step size of 0.1 eV and 0.5 eV were applied for core level scans and survey spectra, respectively. XPS spectra calibration was based on the C-C component in the C1s XPS spectrum at 284.8 eV. The XPS spectra were deconvoluted by fitting a Shirley background and symmetric Voigt function which is listed for each sample in Table S3. EDX measurements were conducted with a Crossbeam 550 scanning electron microscope (Zeiss, Germany) at an applied voltage of 15 kV. SEM images were acquired with the Zeiss Crossbeam 550 SEM at an applied voltage of 2 kV and a current of 10 pA. Prior to taking cross-section SEM images, the Al backsides of the AAO templates were removed by etching in a saturated CuCl_2_/HCl solution by defining the Al area with a Kapton mask featuring a circular hole at the AAO position.

### Photocatalytic characterization

Photocatalytic characterization was carried out by analyzing the photocatalytic degradation of methylene blue (MB) as a model pollutant of water. A sample was mounted into a home-built photocatalysis cell composed of polyether ether ketone (PEEK) and a soda-lime glass window. The sample was exposed to a MB solution (2.5 mg l^-1^, 8 ml) which also contained H_2_O_2_ (200 mM) and kept in darkness for one hour to establish the adsorption-desorption equilibrium of the molecules at the samples surface. Subsequently, the system was irradiated with UV-visible light generated by an Euromex LE.5211 lamp equipped with a Philips 64,230 FO halogen bulb. The absorbance of the dye solution was analyzed every five minutes by UV/Vis spectroscopy. For this, 1 ml of the MB solution was pipetted from the photocatalysis cell into a cuvette, analyzed in the spectrometer, and pipetted back. During that time, illumination of the photocatalysis cell was blocked to prevent degradation within this analysis period. Lambert-Beer’s law was applied to calculate the MB concentration based on the measured absorbance at every point in time. The photocatalytic reaction is assumed to occur *via* the Langmuir-Hinshelwood mechanism. Since the MB concentration is lower than 10^− 3^ mol l^-1^, the reaction rate can be simplified to a pseudo-first-order kinetics law:

$$\:\text{ln}\left(\frac{c}{{c}_{0}}\right)=-k\cdot\:t$$

Here, *c* denotes the concentration of the MB solution at a given time *t*, *c*_0_ describes the concentration at the beginning of the measurement (*t* = 0 h), and *k* is the apparent photocatalytic rate constant which is a measure for the photocatalytic activity of a sample. The photocatalytic MB degradation measurement was repeated three times per sample to monitor changes of the activity over consecutive measurements.

## Electronic supplementary material

Below is the link to the electronic supplementary material.

Supplementary Material 1

## Data Availability

The datasets generated during and/or analyzed during the current study are available from the corresponding author on reasonable request.
