# Supplementary material for: Impact of surface treatments on the photocatalytic performance of anodic aluminum oxide templates
Source: Sci Rep. 2025 Apr 29;15:15030. doi: 10.1038/s41598-025-98635-3 (PMC12041583; doi:10.1038/s41598-025-98635-3)
Supplement: Supplementary file 1 — Supplementary Material 1 [file 41598_2025_98635_MOESM1_ESM.docx]

**Supporting Information**

Impact of Surface Treatments on the Photocatalytic Performance of Anodic Aluminum Oxide Templates

Carina Hedrich,^1,2^ Robin R. Petit,^3^ Matthias M. Minjauw,^3^ Anna R. Burson,^1^ Stefanie Haugg,^1^ Kaline P. Furlan,^2^ Christophe Detavernier,^3^ Jolien Dendooven,^3^ Robert H. Blick,^1^ Robert Zierold.^1,^*

^1^ Center for Hybrid Nanostructures, Universität Hamburg, 22761 Hamburg, Germany.

^2^ Hamburg University of Technology (TUHH), Integrated Ceramic-based Materials Systems Group, Denickestraße 15, 21073 Hamburg, Germany.

^3^ CoCooN Group, Department of Solid State Sciences, Ghent University, Krijgslaan 281/S1,

9000 Ghent, Belgium.

* Corresponding author: robert.zierold@uni-hamburg.de

**Table S1.** EDX measurement at the top surface of TiO_2_-coated AAO samples whose surface chemistry was modified with different treatments prior to ALD. The similar Ti contents indicate that these surface treatments have only a minor effect on the subsequent coating by ALD.

| **Surface treatment** | **Element** | **Atomic %** |
| --- | --- | --- |
| as-prepared | O | 63.3 |
|  | Al | 35.3 |
|  | Ti | 1.4 |
| H_2_O_2_ | O | 60.7 |
|  | Al | 37.8 |
|  | Ti | 1.5 |
| H_3_PO_4_ | O | 64.2 |
|  | Al | 34.6 |
|  | Ti | 1.2 |


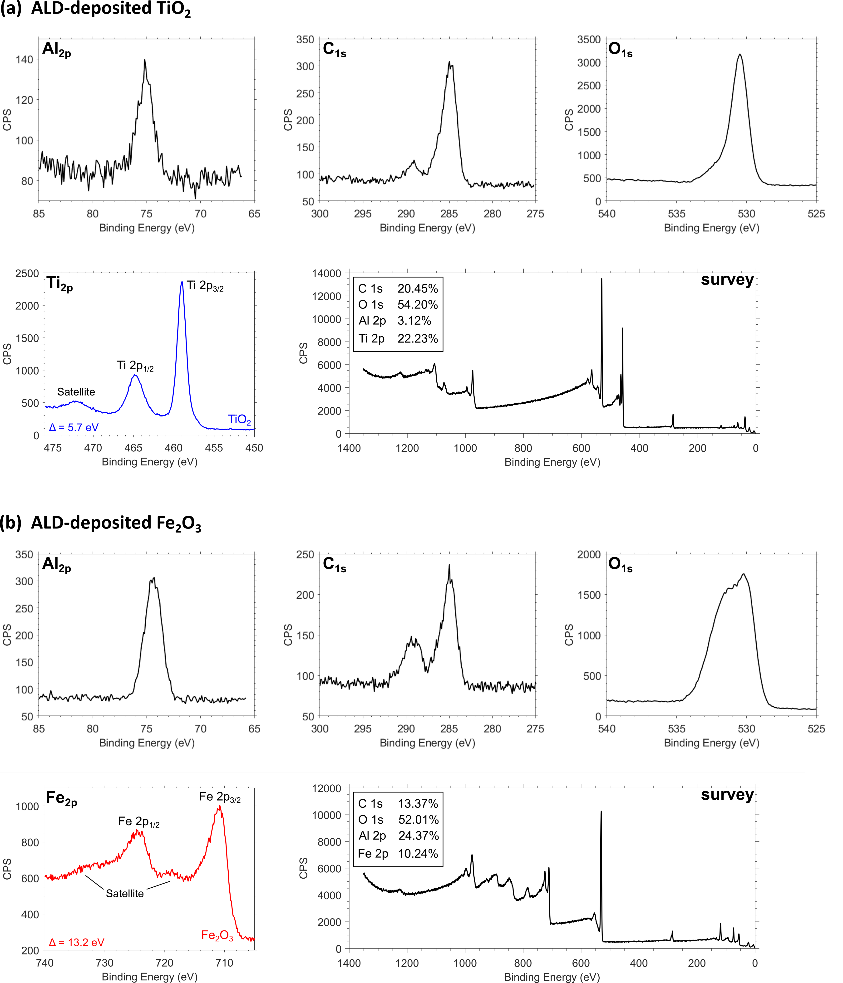


**Figure S1.** XPS spectra of the ALD-grown photocatalysts on AAO structures. (a) TiO_2_ deposition is indicated by the Ti peak spin splitting of 5.7 eV. (b) ALD of Fe_2_O_3_ is identified by 13.2 eV spin splitting, the Fe_2p3/2_ peak at 711 eV B.E. and low intensity of the satellite peaks.


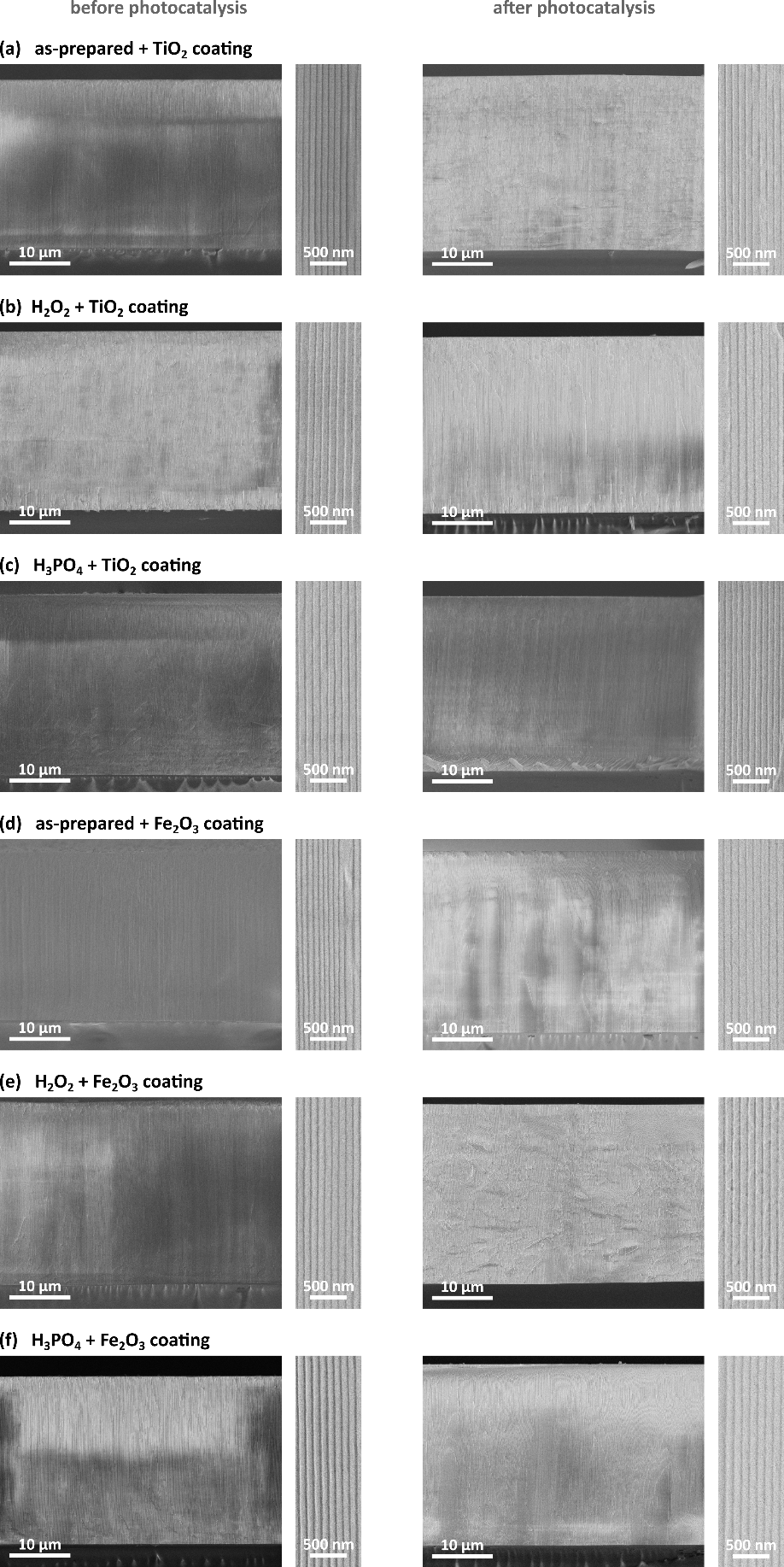


**Figure S2.** Cross-section SEM images of ALD-coated AAO structures that were exposed to different surface treatments prior ALD coating with different photocatalysts – TiO_2_ or Fe_2_O_3_. Comparing the different surface treatments does not reveal an effect on the pore morphology. The same pore morphologies are observed before (left column) and after photocatalysis measurements (right column) for two different batches.


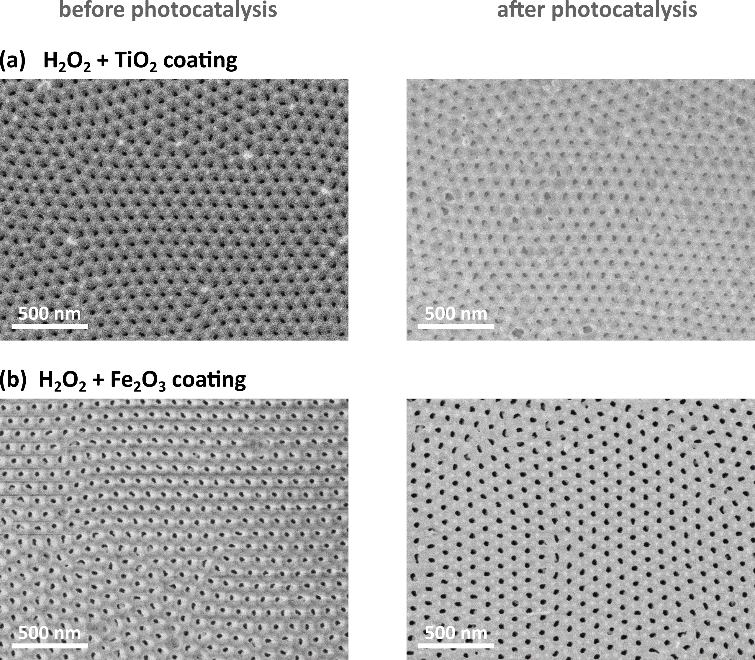


**Figure S3.** Top-view SEM images of H_2_O_2_ surface-treated and ALD-coated AAO structures. After surface treatment, the structures were coated with (a) TiO_2_ and (b) Fe_2_O_3_. The photocatalysis measurements do not provoke significant changes in the pore morphology and ALD-grown photocatalysts as obvious in the comparison of two different sample batches before and after photocatalysis.


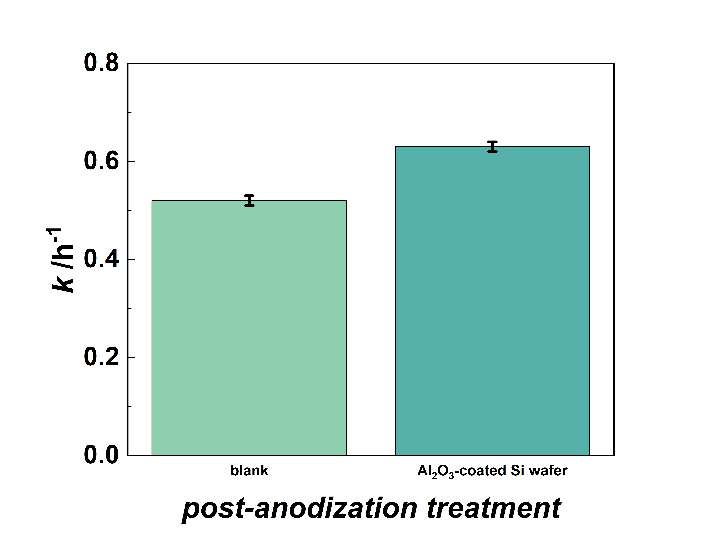


**Figure S4.** Photocatalytic MB degradation measured in the PEEK cell without sample (blank) and with a planar Si wafer coated with 20 nm Al_2_O_3_ by ALD. The measurements reveal degradation rates of 0.52 ± 0.01 h^-1^ for the blank measurement and 0.63 ± 0.01 h^-1^ for the Al_2_O_3_-coated Si wafer. Note that only one measurement was conducted, respectively.

**Table S2.** Identified XPS peak positions of the AAO samples exposed to different post-anodization surface treatments.

| **Peak** | **Assign-ment** | **Ref.** | **As-prepared** | | **Annealed** | | **H_2_O** | | **H_2_O_2_** | | **H_3_PO_4_** | |
| --- | --- | --- | --- | --- | --- | --- | --- | --- | --- | --- | --- | --- |
|  |  |  | **B.E. /eV** | **%Area** | **B.E. /eV** | **%Area** | **B.E. /eV** | **%Area** | **B.E. /eV** | **%Area** | **B.E. /eV** | **%Area** |
| Al_2p_ | Al_2_O_3_ | ^1,2^ | 74.11 | 42.39 | 74.04 | 53.15 | 74.19 | 71.25 | 74.29 | 78.42 | 73.87 | 24.63 |
|  | AlOOH or AlPO_4_ | ^3^ | 75.31 | 31.27 | 74.93 | 25.22 | 75.06 | 20.29 | 75.31 | 21.58 | 74.91 | 27.58 |
|  | Al-O | ^4,5^ | 76.62 | 26.34 | 76.55 | 11.15 | 76.60 | 4.55 |  |  | 76.20 | 29.05 |
|  | AlF_3_ | ^2^ |  |  | 77.55 | 10.47 | 77.56 | 3.91 |  |  | 77.19 | 18.73 |
| C_1s_ | C-C | ^2,6–9^ | 284.66 | 29.86 | 284.70 | 44.54 | 284.76 | 56.12 | 284.68 | 50.18 | 284.23 | 18.69 |
|  | C-OH or C=C | ^8,9^ | 285.63 | 19.18 | 285.63 | 16.39 | 285.63 | 8.86 | 285.63 | 20.47 | 285.48 | 19.87 |
|  | C-O or C-O-P | ^1,2,6,9^ | 286.78 | 19.09 | 286.84 | 6.48 | 286.78 | 7.07 | 286.78 | 4.39 | 286.87 | 21.97 |
|  | C=O | ^7,9^ | 288.01 | 9.40 | 287.95 | 11.86 | 288.40 | 11.12 | 288.57 | 6.99 | 288.28 | 9.23 |
|  | O-C=O/ oxalate | ^1,2,10^ | 289.32 | 10.32 | 289.21 | 10.45 | 289.47 | 13.69 | 289.44 | 12.35 | 289.76 | 9.65 |
|  | C-F | ^8^ | 290.64 | 7.94 | 290.56 | 7.38 | 290.93 | 3.14 | 290.60 | 5.62 | 290.94 | 9.20 |
|  | CF_3_ | ^8^ | 292.03 | 3.37 | 292.47 | 16.39 |  |  |  |  | 292.18 | 7.48 |
| O_1s_ | Al_2_O_3_ | ^2,3^ | 531.10 | 32.64 | 531.13 | 59.50 | 531.05 | 46.26 | 531.13 | 46.29 | 531.05 | 34.11 |
|  | OH groups | ^3,5^ | 532.55 | 46.55 | 532.83 | 28.76 | 532.44 | 48.31 | 532.45 | 53.71 | 532.65 | 41.64 |
|  | COOH/ H_2_O | ^6,9,10^ | 534.11 | 20.81 | 534.68 | 11.75 | 534.60 | 5.43 |  |  | 534.28 | 24.25 |
| P_2p_ | PO_4_^3-^ | ^1,3^ |  |  |  |  |  |  |  |  | 133.85 | 33.88 |
|  | P-O | ^4,9^ |  |  |  |  |  |  |  |  | 134.72 | 16.93 |
|  | P_2p 3/2_ | ^1^ |  |  |  |  |  |  |  |  | 135.95 | 32.27 |
|  | P-F | ^2,4^ |  |  |  |  |  |  |  |  | 136.82 | 16.92 |

**Table S3**. Fitting parameters of the XPS peaks.

| **Sample** | **Peak** | **FWHM** | **L.Sh.** |
| --- | --- | --- | --- |
| as-prepared | Al_2p_ | 1.60 | LA(50) |
|  | C_1s_ | 1.60 | LA(50) |
|  | O_1s_ | 2.00 | LA(50) |
| annealed | Al_2p_ | 1.54 | LA(50) |
|  | C_1s_ | 1.54 | LA(50) |
|  | O_1s_ | 2.18 | LA(50) |
| H_2_O | Al_2p_ | 1.56 | LA(50) |
|  | C_1s_ | 1.57 | LA(50) |
|  | O_1s_ | 2.05 | LA(50) |
| H_2_O_2_ | Al_2p_ | 1.62 | LA(50) |
|  | C_1s_ | 1.42 | LA(50) |
|  | O_1s_ | 2.09 | LA(50) |
| H_3_PO_4_ | Al_2p_ | 1.59 | LA(50) |
|  | C_1s_ | 1.60 | LA(10) |
|  | O_1s_ | 2.00 | LA(50) |
|  | P_2p_ | 2.07 | LA(50) |


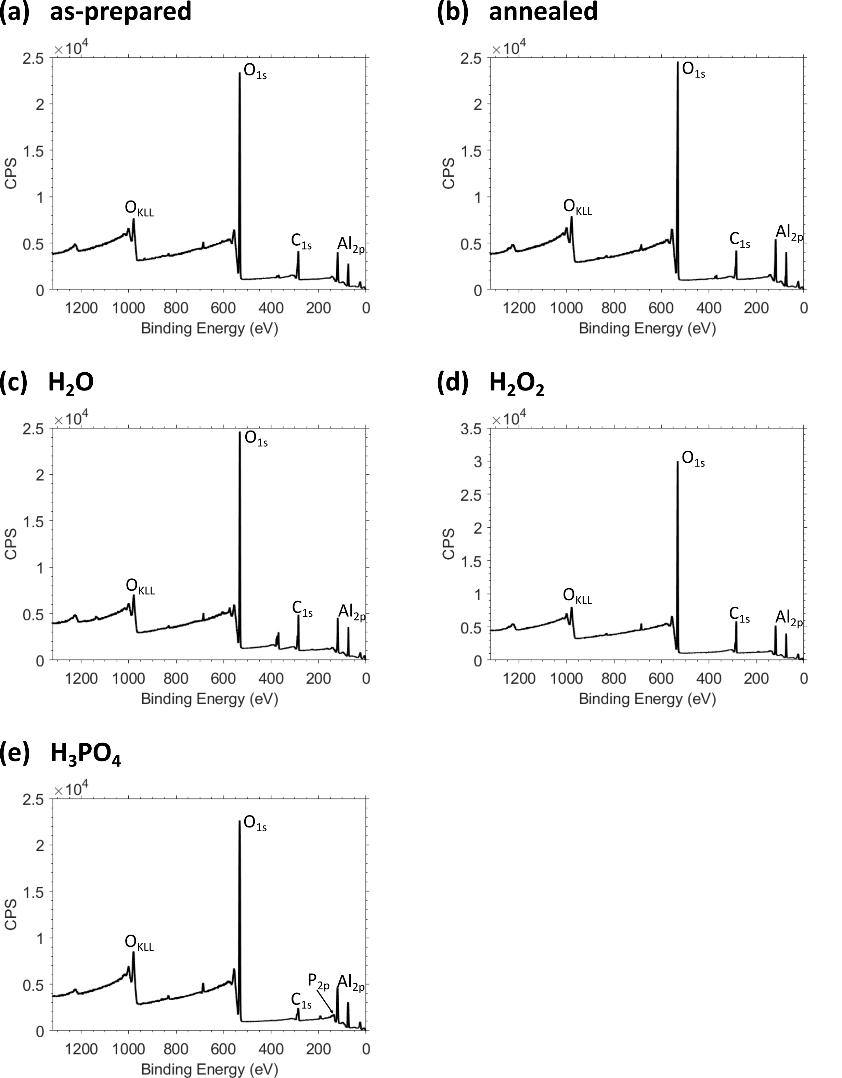


**Figure S5.** XPS survey scans of surface modified AAO structures. The AAO samples are (a) as-prepared, (b) annealed at 450 °C for 1 h, (c) exposed to H_2_O for 24 h, (d) immersed into H_2_O_2_ for 24 h and H_2_O for additional 24 h, and (e) immersed in 5 wt% H_3_PO_4_ for 1 min. hh


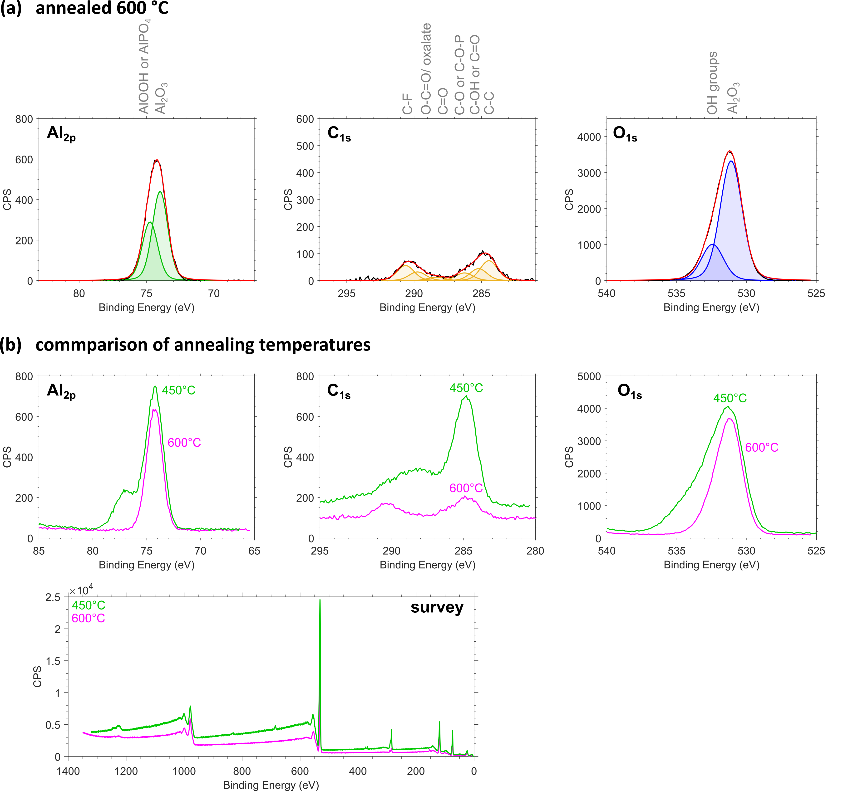


**Figure S6.** XPS measurements of a AAO structure thermally annealed at 600 °C for 1 h (a) show a reduced -OH group and carbon content compared to the structure annealed at 450 °C (b).

**Table S4.** Identified XPS peak positions and fitting parameters of the AAO structure annealed at 600 °C.

| **Peak** | **Assign-ment** | **Ref.** | **Annealed 600 °C** | | **FWHM** | **L.Sh.** |
| --- | --- | --- | --- | --- | --- | --- |
|  |  |  | **B.E. /eV** | **%Area** |  |  |
| Al_2p_ | Al_2_O_3_ | ^1,2^ | 74.00 | 60.33 | 1.35 | LA(50) |
|  | AlOOH or AlPO_4_ | ^3^ | 74.75 | 39.67 | 1.35 | LA(50) |
|  | Al-O | ^4,5^ |  |  |  |  |
|  | AlF_3_ | ^2^ |  |  |  |  |
| C_1s_ | C-C | ^2,6–9^ | 284.45 | 29.90 | 1.50 | LA(50) |
|  | C-OH or C=C | ^8,9^ | 285.22 | 17.95 | 1.50 | LA(50) |
|  | C-O or C-O-P | ^1,2,6,9^ | 286.26 | 11.52 | 1.50 | LA(50) |
|  | C=O | ^7,9^ | 288.35 | 5.10 | 1.50 | LA(50) |
|  | O-C=O/ oxalate | ^1,2,10^ | 289.75 | 12.99 | 1.50 | LA(50) |
|  | C-F | ^8^ | 290.63 | 22.54 | 1.50 | LA(50) |
|  | CF_3_ | ^8^ |  |  |  |  |
| O_1s_ | Al_2_O_3_ | ^2,3^ | 531.10 | 76.75 | 1.86 | LA(50) |
|  | OH groups | ^3,5^ | 532.43 | 23.25 | 1.86 | LA(50) |
|  | COOH/ H_2_O | ^6,9,10^ |  |  |  |  |


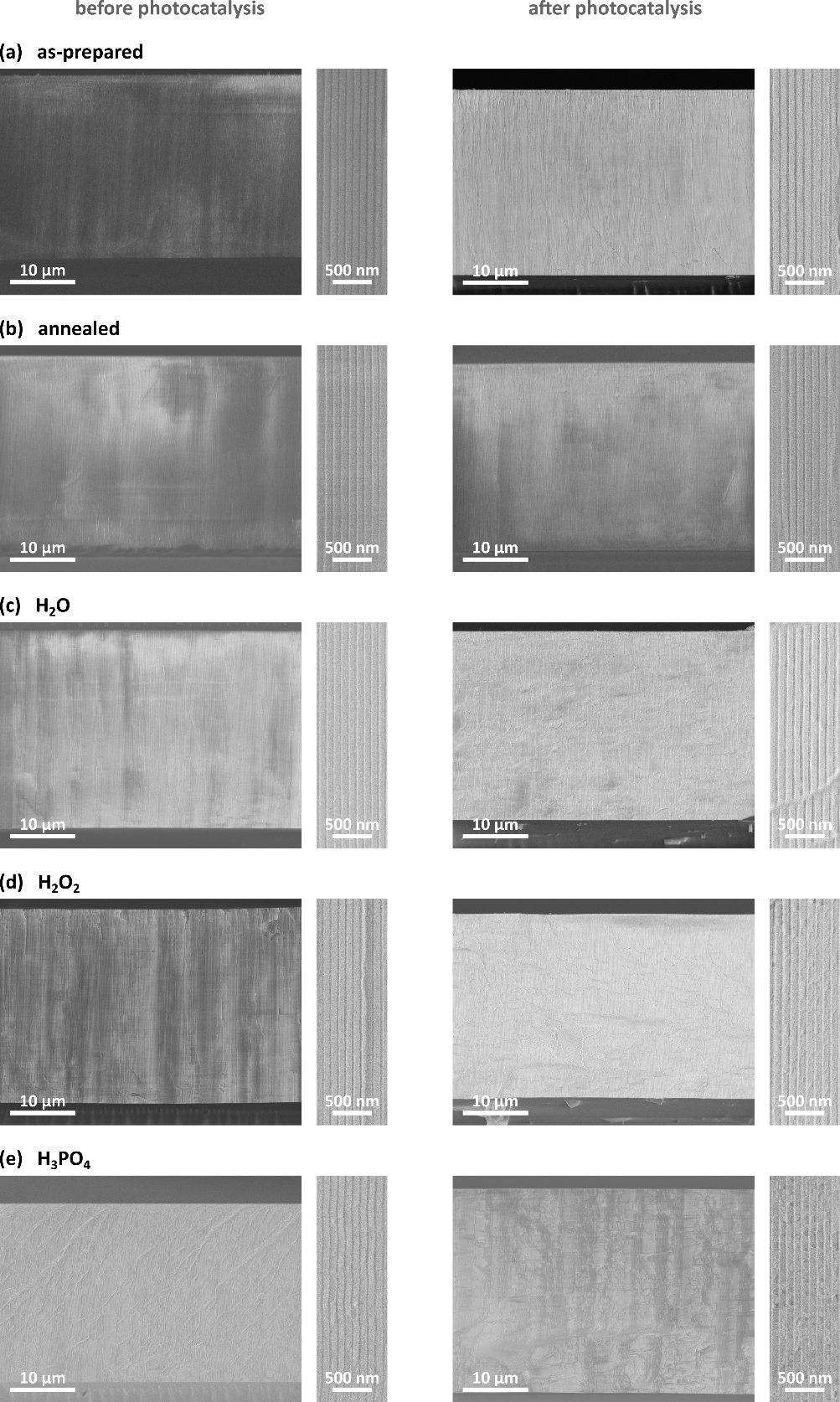


**Figure S7.** Cross-section SEM images of AAO templates with different surface treatments before (left column) and after photocatalysis (right column) of two different batches. Neither the surface treatments nor the photocatalysis measurements affect the pore morphology.


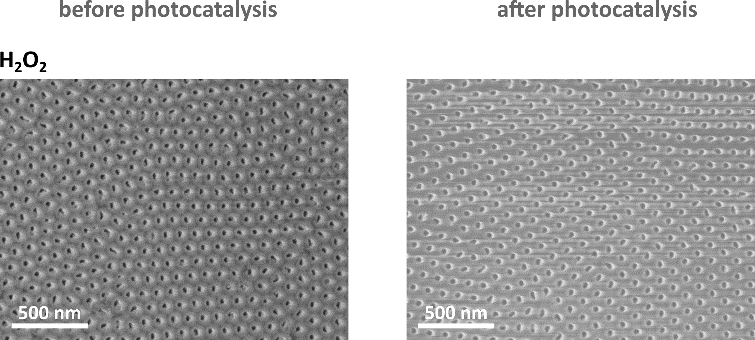


**Figure S8.** Top-view SEM images of a H_2_O_2_-treated AAO structure before and after photocatalysis measurement of two different batches. The AAO structure morphology is not affected by the photocatalysis measurement.

References

1. Verdier, S. *et al.* XPS Study on Al_2_O_3_- and AlPO_4_-Coated LiCoO_2_ Cathode Material for High-Capacity Li Ion Batteries. *J. Electrochem. Soc.* **154**, A1088 (2007).

2. Baggetto, L., Dudney, N. J. & Veith, G. M. Surface chemistry of metal oxide coated lithium manganese nickel oxide thin film cathodes studied by XPS. *Electrochimica Acta* **90**, 135–147 (2013).

3. Andreeva, R., Stoyanova, E., Tsanev, A. & Stoychev, D. Influence of the Pre-Treatment and Post-Treatment Operations on the Surface Chemistry and Corrosion Behavior of Cerium-Based Conversion Coatings on Aluminum. in *Current Advances in Chemistry and Biochemistry Vol. 7* (ed. Romero, Dr. A. M.) 1–28 (Book Publisher International (a part of SCIENCEDOMAIN International), 2021). doi:10.9734/bpi/cacb/v7/8429D.

4. Deguchi, M., Todorov, Y. M. & Abe, K. Functional electrolyte: Design of anti-corrosion additives for Al collectors in LiFSI-based electrolyte. *Electrochimica Acta* **469**, 143267 (2023).

5. Li, X. *et al.* Influence of oxygen pressure and substrate temperature on the properties of aluminum fluoride thin films. *Appl. Surf. Sci.* **282**, 226–230 (2013).

6. Chenakin, S. & Kruse, N. XPS characterization of transition metal oxalates. *Appl. Surf. Sci.* **515**, 146041 (2020).

7. Morais, A., Alves, J. P. C., Lima, F. A. S., Lira-Cantu, M. & Nogueira, A. F. Enhanced photovoltaic performance of inverted hybrid bulk-heterojunction solar cells using TiO_2_ reduced graphene oxide films as electron transport layers. *J. Photonics Energy* **5**, 057408 (2015).

8. Peng, Y. *et al.* Air plasma-induced carbon fluoride enabling active C F bonds for double-high energy/power densities of Li/CFx primary battery. *J. Alloys Compd.* **905**, 164151 (2022).

9. Singh, A. S., Advani, J. H. & Biradar, A. V. Phosphonate Functionalized Carbon Spheres as a Brønsted Acid Catalysts for Valorization of bio-renewable α-Pinene Oxide to trans-Carveol. *Dalton Trans.* **49**, 7210 (2020).

10. Chenakin, S. P., Szukiewicz, R., Barbosa, R. & Kruse, N. Surface analysis of transition metal oxalates: Damage aspects. *J. Electron Spectrosc. Relat. Phenom.* **209**, 66–77 (2016).
